# Prevalence and risk factors of stroke-related sarcopenia at the subacute stage: A case control study

**DOI:** 10.3389/fneur.2022.899658

**Published:** 2022-08-08

**Authors:** Ruihong Yao, Liqing Yao, Amin Rao, Jibing Ou, Wenli Wang, Qinzhi Hou, Chunyan Xu, Bu-Lang Gao

**Affiliations:** ^1^Medical Imaging Department, The First Affiliated Hospital of Kunming Medical University, Kunming, China; ^2^Rehabilitation Medicine Department, The Second Affiliated Hospital of Kunming Medical University, Kunming, China

**Keywords:** sarcopenia, stroke-related, prevalence, stroke, rehabilitation

## Abstract

**Purpose:**

To investigate the prevalence and risk factors of stroke-related sarcopenia (SRS) in hospitalized patients receiving rehabilitation treatment.

**Methods:**

Approximately, 259 patients with stroke that satisfied the inclusion and exclusion criteria were consecutively recruited between June 2020 and July 2022. The epidemiologic data, history, clinical data, and measured data of the skeletal muscle index were collected. The patients were divided into the sarcopenia and non-sarcopenia group for comparison and analysis with the univariate and multivariate analysis.

**Results:**

SRS was presented in 121 (46.7%) patients with a mean age of 59.6 ± 9.7 years, including 42 women and 79 men. Multivariate logistic regression analysis revealed the following parameters to be significant (*p* < 0.05) risk factors for SRS: college degree or above (OR, 2.1, 95% CI, 1.1–4.1), ICU stay (OR, 1.7, 95% CI, 1.06–2.8), pneumonia (OR, 1.9, 9% CI, 1.1–3.6), walking ability (OR, 2.6, 95% CI, 1.5–4.6), cognitive impairment (OR, 1.8, 95%, 1.1–2.9), aphasia (OR, 2.1, 95% CI, 1.2–3.5), nasogastric feeding (OR, 3.7, 95%, 1.9–7.3), age (OR, 1.04, 95% CI, 1–1.1), and creatine kinase (OR, 1.1, 95% CI,0.9–1.2).

**Conclusions:**

Older age, light weight, severer clinical conditions, cognitive impairment, and significantly decreased levels of albumin, RAG, creatinine, uric acid, red blood cell count, hemoglobin, prealbumin, iron, and creatine kinase are more significantly present in patients with SRS compared with those without SRS.

## Introduction

Sarcopenia is the loss of skeletal muscle mass and strength with aging and has become a worldwide social issue with an increased risk of adverse outcomes, including falls, fractures, longer hospitalization duration, physical disability, and mortality (1–4). The prevalence of sarcopenia reported in the literature varies with sex, age, race, and diagnostic criteria of sarcopenia (5), ranging from 15 to 50% in older adults (6), 3.1 to 29% in community dwelling residents (7), and 14–33% in patients receiving long-term care (8, 9). Sarcopenia usually has some common risk factors with some diseases, such as heart failure, cardiovascular disease (CVD), renal dialysis, fracture, diabetes mellitus (DM), and chronic obstructive pulmonary disease (COPD), which harbor the following common features, including physical inactivity, older age, malnutrition, inflammation or endocrine disorders (10). It has been reported that the prevalence of sarcopenia was 31.4, 31.1, and 26.8% in CVD, DM, and COPD, respectively (11). The loss of skeletal muscle mass and strength in patients with stroke is called stroke-related sarcopenia (SRS) (12, 13). The risk factors in primary sarcopenia include gender, age, and levels of exercise; however, the risk factors in SRS are unknown and cannot be prevented effectively. At the same time, the prevalence of SRS is not clear either. Both stroke and sarcopenia mainly happen in patients of more than 60 years of age. There are approximately 2.5 million cases of new stroke every year in China, and more than 60% of the patients with stroke remain disabled and need rehabilitation therapy, with 50% of patients suffering from hemiparesis and 30% unable to walk without assistance (14, 15). However, in rehabilitation therapy, most physicians care about the recovery of limb motor function but ignore the impact of sarcopenia on rehabilitation therapy because sarcopenia may prolong the recovery process. In sarcopenia, a combination of disuse, denervation, remodeling, inflammation, and spasticity accounts for a complex pattern of muscle tissue phenotype change and atrophy (12). SRS decreases the treatment effect and affects the quality of life of the patients, which has not been recognized in the guidelines for rehabilitation treatment of sarcopenia (16). Currently, the prevalence, risk factors, and clinical features of SRS in hospitalized patients with stroke are unknown, and it was hypothesized that knowledge of the above information would facilitate implementation of effective measures to prevent SRS and promote the recovery and rehabilitation of these patients. In order to assess the prevalence, risk factors, and clinical characteristics of SRS, patients with stroke were recruited and evaluated in this study with the standard of skeletal muscle mass measurement by bioelectrical impedance analysis (BIA) according to the 2019 consensus update on sarcopenia diagnosis and treatment of the Asian Working Group for sarcopenia (17).

## Materials and methods

### Study design and participants

All methods were carried out in accordance with relevant international guidelines and regulations. This single-center, observational, case control study was approved by the ethics committee of a tertiary academic hospital (Kunming, China, and approval No.: FEY-BG-39–2.0), and all patients had provided written informed consent to participate. Patients with consecutive stroke without sarcopenia after stroke assessed by the SARC-F score hospitalized in the Rehabilitation Medicine Department of the Second Affiliated Hospital of Kunming Medical University between June 2020 and July 2022 were enrolled based on skeletal muscle mass and strip strength according to the interpretation of Asian Working Group for sarcopenia: 2019 consensus update on sarcopenia diagnosis and treatment (17). The inclusion criteria were patients with hemorrhagic or ischemic stroke for the first time, <6 months from the stroke onset to being hospitalized for rehabilitation, 18–80 years of age, hemiplegia, grip strength measurable on one side, and the National Institute of Health Stroke Scale (NIHSS) ≤ 12 points. The patients were excluded if they had a history of heart, kidney, and lung failure, cardiovascular disease (CVD), renal dialysis, fracture, osteoarthritis, diabetes mellitus (DM), chronic obstructive pulmonary disease (COPD), transient ischemic attack (TIA), pregnancy, coma, and quadriplegia.

### Study consent and measurement

The basic information was collected, including age, gender, duration time, time of the onset of stroke, stroke type, marriage state, history of cognitive impairment, coma, aphasia, pneumonia, hypertension, diabetes, smoking status, alcohol consumption, thrombolysis, and the level of physical activity, such as the SARC-F score. Stroke was classified as ischemic and hemorrhagic according to the computed tomography (CT)/magnetic resonance imaging (MRI) data. The blood test was performed on the next day after fast for 8 h, including total triglycerides (TG), total protein (TP), albumin (Alb), hemoglobin (HGB), creatinine, and blood urea nitrogen. Height, weight, and body mass index (BMI) were measured on the 1st day of hospitalization. The stroke type of all the patients had been classified by Bamford classification, and the neurological function was evaluated with NIHSS.

### Diagnosis of sarcopenia, anxiety, and depression

Sarcopenia was defined as presentation of both decreased muscle mass and strength. The patients were tested in the supine or sitting position using a BIA instrument (Inbody s10; Inbody, KOREA). Skeletal muscles of the arm and the leg on the affected and non-affected sides were measured. The cut-off value of height-adjusted skeletal muscle index (SMI) for men and women is <7. kg/m^2^ and <5.7 kg/m^2^, respectively, and handgrip strength was <28 kg in men and 18 kg in women as the sarcopenia diagnosis standard. In light of the Asian Working Group for Sarcopenia, SMI was calculated based on the skeletal muscle mass by the height squared, and handgrip strength was measured by a Jamar electronic grip hand dynamometer (Javar, Patterson, USA) in kilograms three times, with the highest measurements being recorded. The greatest of three measurements obtained from the non-hemiplegia hand was used for analysis. The patients were in a standing, lying, or sitting position with the arms put straight by the sides, depending on the patients' motor ability.

The SARC-F scores with a simple five-item questionnaire were applied to evaluate the muscle strength and physical function changes, ranging from 0 to 10, with 0–2 points for each component (0 = best and 10 = worst) (18). The five items were strength, walking, rising from a chair, climbing ten stairs, and falls. Every item had three scores, with 0 standing for no difficulty to do it, 1 for a little difficulty, and 2 for a lot of difficulty or inability or use of aids to do it. The SARC-F scale was a simple, rapid, and effective approach to assess the physical function and balance problems.

Body weight and height were measured by using a standardized electronic scale to the nearest recision scale of 0.1 kg and 0.01 m, with the subjects wearing light clothes without shoes and socks. The BMI was calculated (kg/m^2^).

Two psychiatrists independently assessed anxiety and depression with the Hamilton Anxiety Scale (HAMA) and Hamilton Depression Scale (HAMD) 1 week after being hospitalized (19, 20).

### Sample size calculation

The sample size was calculated using the approach from the literature (21). Based on the mean difference of SMI in patients with stroke with and without sarcopenia, the standard deviation was 2.65, which indicated a sample size of at least 104 participants in each group to reject the null hypothesis, with a power of 0.8 and an alpha error of 0.05. The sample size of 259 patients would satisfy the requirement.

### Statistical analysis

All analyses were performed using the IBM SPSS software (version 23, Armonk, NY, USA). Continuous measurement data in normal distribution were presented as mean ± standard deviation (SD), and categorical data were presented as numbers and frequencies (%). The statistical significance of differences between groups was assessed using an independent sample *t*-test for continuous normal variables and the chi-square test for categorical variables. Multivariate logistic regression analyses were used to calculate the odds ratios (ORs) and 95% confidence interval (CI) for the effects of risk factors on the presence of sarcopenia. Binary and multivariate logistic regression analyses were used to determine whether the presence of sarcopenia on hospital admission was independently associated with different parameters. *p* < 0.05 was considered to be statistically significant.

## Results

A total of 259 patients with stroke were enrolled, including 179 men and 80 women (Tables 1–4). SRS was presented in 121 (46.7%) patients with a mean age of 59.6 ± 9.7 (range, 18–85) years, including 42 women (16.2%; mean age, 61.7 ± 16.6 years) and 79 men (30.5%; mean age, 55.9 ± 13.3 years). The other 138 (53.3%) patients had no SRS with an age range of 22–88 (mean, 53.3 ± 13.9) years, including 38 women (14.7%) and 100 men (38.6%). The two groups had similar duration after the onset of stroke: 45 (28–78) vs. 40 (22.5–72.3) days for patients with SRS and patients without SRS, respectively (Table 2). The SARC-F score was 7.9 (2–10) in patients with SRS, which was significantly (*p* = 0.001) greater than that of 6.4 (0–10) in patients without SRS (Table 1), and the sarcopenia was more prominent in the affected side of stroke than on the other side in patients with SRS or without SRS.

**Table 1:** Categorical data of patients with and without sarcopenia (*n*, %).

	**Total**	**Sacopenia**	**Non-Sarcopnia**	**P**	**OR**	**95%CI**	**P**
Number	259	121	138				
Male	179 (68.9%)	79 (65%)	100 (73.4%)	0.14			
Married	225 (87.1%)	104 (85.7%)	122 (88.7%)	0.47			
Han nationality	215 (83.0%)	100 (82.9%)	114 (83.1%)	0.96			
Hemorrhagic stroke	136 (52.7%)	64 (53.6%)	72 (51.6%)	0.75			
Infarct	123 (47.5%)	57 (47.1%)	66 (49.3%)	0.63			
With coma history	150 (58.7%)	68 (56.4%)	83 (61.3%)	0.18			
Tracheotomy history	65 (25.4%)	36 (31.4%)	26 (18.5%)	0.05			
College degree or above	47 (18.2%)	16 (12.9%)	33 (24.2%)	0.02	2.1	1.1–4.1	0.02
Smoke history	116 (44.7%)	54 (45%)	61 (44.4%)	0.9			
Alcohol abuse	86 (33.3%)	41 (33.6%)	46 (33.1%)	0.9			
History of brain surgery	109 (42.4%)	54 (45%)	55 (39.5%)	0.5			
History of thrombolysis	47 (17.8%)	24 (20%)	21 (15.3%)	0.44			
Hypertension	171 (65.9%)	80 (67.2%)	89 (64.5%)	0.7			
Diabetes mellitus	68 (27.3%)	33 (29.3%)	34 (25%)	0.4			
History in ICU	130 (50.8%)	69 (57.1%)	60 (43.5%)	0.03	1.7	1.06–2.8	0.03
Gout	23 (9.1%)	8 (7.1%)	15 (11.3%)	0.3			
Pneumonia history	75 (28.8%)	46 (37.9%)	19 (18.5%)	0.001	1.9	1.1–3.6	0.03
Bone fracture	39 (14.8%)	17 (15%)	20 (14.5%)	0.9			
Ability of walking	93 (36%)	28 (22.9%)	70 (50.8%)	0	2.6	1.5–4.6	0.001
Cognitive impairment	113 (43.6%)	65 (54.3%)	44 (31.5%)	0	1.8	1.1–2.9	0.001
Aphasia	123 (47.3%)	69 (57.1%)	50 (36.3%)	0.001	2.1	1.2–3.5	0.007
Depression	57 (21.6%)	25 (20.7%)	31 (22.6%)	0.71			
Anxiety	35 (13.6%)	14 (11.4%)	19 (16.1%)	0.3			
Nasogastric feeding	158 (61.0%)	91 (75%)	54 (45.2%)	0	3.7	1.9–7.3	0

*OR, odds ratio; CI, confidence interval; ICU, intensive care unit*.

**Table 2:** Continuous data of patients with and without sarcopenia.

	**Total**	**Sarcopenia**	**Non–sarcopenia**	**P**	**OR**	**95% CI**	**P**
No.	259	121 (46.7%)	138 (53.3%)				
F/M	80/179	42/79	38/100	0.14			
Height,m	1.7 (1.6–1.7)	1.7 (1.6–1.7)	1.7 (1.6–1.7)	0.193			
Weight,kg	63.8 ± 10.2	59.6 ± 9.7	68.58.5	0.000	0.88	0.85–0.92	0
Age,y	57 (18–88)	59 (18–85)	53 (22–88)	0.000	1.04	1.0–1.1	0
SARC–F	7.2 (0–10)	7.9 (2–10)	6.4 (0–10)	0.001			
NIHSS	9.2 (3–15)	12.7 (8–15)	6.5 (3–10)	0.013	1.78	1.21–3.62	0
LSMAS,kg	8.5 (7.7–9.8)	6.5 (5.1–10.7)	10.5 (9.2–12.1)	0.039			
LSMNAF,kg	11.2 (8.6–13.6)	7.8 (6.2–11.3)	11.7 (10.5–14.3)	0.051			
Recovery time,d	11 (2.0–37.0)	10 (1–28)	13 (2–45)	0.08			
Duration time,d	42 (28.0–73.0)	45 (28–78)	40 (22.5–72.3)	0.484			
Cognitive impairment	26.5 (22.1–28.3)	23.3 (21.0–27.1)	27.5 (25.4–29.1)	0.001	1.7	1.1–2.9	0.001
depression	9.5 (7.2–15.6)	13.5 (9.5–17.6)	11.7 (8.4–16.8)	0.061			
anxiety	10.3 (7.5–13.7)	12.8 (7.2–14.2)	10.9 (5.6–13.9)	0.058			
Left upper limb,cm	29 (27.0–31.0)	27 (25–29)	31 (29–33)	0.000			
Right upper limb,cm	30 (27.0–31.0)	27 (25–29)	31 (30–33)	0.000			
Left grip,kg	3.3 (0.0–14.0)	9.5 (0–20.4)	2.0 (0–10)	0.000			
Right grip,kg	2 (0.0–14.0)	1.0 (0–8.0)	6.5 (0–20.0)	0.000			
Left CC,cm	31.2 ± 3.3	28.7 ± 1.9	34.0 ± 2.2	0.000			
Right CC,cm	31.3 ± 3.4	29.2 ± 2.0	35.0 ± 2.4	0.000			
Total protein,g/L	65.3 (62.1–68.7)	65.8 (61.7–68.5)	65.2 (62.5–68.9)	0.48			
Albumin,g/L	37.4 (34.9–39.6)	36.2 (33.9–38.9)	38.1 (36.4–40.4)	0.000	0.89	0.82–0.97	0.007
RAG	1.35 ± 0.2	1.3 ± 0.2	1.4 ± 0.2	0.000	0.12	0.04–0.4	0
Glucose,mmol/L	5.43 (4.9–6.3)	5.4 (4.8–6.8)	5.4 (4.9–6.1)	0.578			
Urea,mmol/L	5 (4.0–6.5)	5.0 (3.8–6.3)	5.1 (4.1–6.5)	0.35			
Creatinine,umol/L	60 (49.0–75.0)	56 (47–67)	66 (54–82.8)	0.000	0.994	0.989–1.0	0.04
Uric acid,umol/L	317 (253.0–390.0)	291 (226–368)	349 (285–423)	0.000	0.996	0.993–0.998	0.02
Red blood cell,10–12/L	4.4 ± 0.7	4.2 ± 0.7	4.6 ± 0.6	0.000	0.463	0.3–0.7	0
Hemoglobin,g/L	131.1 ± 18.9	126 ± 20.1	136 ± 15.9	0.000	0.968	0.954–0.982	0
Prealbumin,mg/L	239 (195.0–278.0)	212 (177–259)	255 (225–302)	0.000	0.99	0.986–0.994	0
Total cholesterol,mmol/L	3.78 (3.1–4.5)	3.8 (3.2–4.5)	3.8 (3.1–4.5)	0.764			
Triglyceride,mmol/L	1.34 (1.0–1.8)	1.3 (1.0–1.7)	1.4 (1.1–2.0)	0.09			
HDL,mmol/L	0.94 (0.8–1.1)	0.92 (0.78–1.1)	0.94 (0.8–1.1)	0.808			
LDL,mmol/L	2.25 (1.7–2.9)	2.3 (1.8–2.9)	2.2 (1.7–3.0)	0.837			
NHDLC,mmol/L	2.8 (2.2–3.5)	2.8 (2.2–3.5)	2.7 (2.1–3.5)	0.781			
Iron,umol/L	12 (8.8–15.5)	10.5 (7–14.5)	14.2 (10.1–16.8)	0	0.93	0.89–0.98	0
LDH,U/L	167 (144.0–201)	174 (149–211)	163 (140–187.5)	0.013			
ALT,U/L	23 (15.0–40)	23 (15–40)	24.5 (16.3–39.8)	0.355			
Creatine kinase,U/L	48 (28.0–75)	36 (21–68)	59 (38.5–86.3)	0.000	1.1	0.9–1.2	0.325

*OR, odds ratio; CI, confidence interval; CC, calf circumstance; RAG, Ratio of albumin to globulin; HDL, high–density lipoprotein; LDL, low–density lipoprotein; NHDLC, non–high–density lipoprotein cholesterol; LDH, lactate dehydrogenase; ALT, Alanine aminotransferase. Recovery time means the total rehabilitation time. Disease course means the duration time from the stroke onset to the time of this study; LSMAS, Limb skeletal muscle of affected side; LSMNAS, Limb skeletal muscle of non–affected side. Data presentation: If the continuous data conformed to normal distribution, mean ± SD (standard deviation) was used. If the continuous data did not conform to normal distribution or were in skew distribution, the data were presented as median and interquartile (a 25–75% range)*.

**Table 3:** Clinical characteristic of patients with SRS and patients with non–SRS.

	**Female(*****n*** = **80)**				**Male (*****n*** = **179)**			
	**Total**	**SRS**	**Non–SRS**	**P**	**Total**	**SRS**	**Non–SRS**	**P**
No.	80	42 (52.5%)	38 (47.5%)	0	179	79 (44.1%)	100 (55.9%)	0
Age,y	61.7 ± 16.6	64.4 ± 16.9	58.8 ± 16.1	0.136	56.0 ± 13.7	59.73 ± 12.8	53.1 ± 13.7	0.001
BMI,kg/m^2^	22.97 ± 3.9	21.0 ± 3.4	25.2 ± 3.3	0	23.1 ± 3.2	21.6 ± 3.1	24.2 ± 2.8	0
Duration,d	38.5 (22.0–67.5)	47.0 (26.3–101.8)	29.5 (20.8–57.0)	0.086	51.5 (30–87)	50.0 (30.0–96.0)	53.0 (30.0–82.0)	0.683
Glucose,mmol/L	5.3 (4.8–5.9)	5.2 (4.7–6.0)	5.3 (4.9–7.2)	0	5.2 (4.8–5.8)	5.0 (4.7–5.7)	5.3 (4.8–5.9)	0.167
Insulin,IU	9.9 (6.4–14.1)	8.2 (5.4–12.0)	11.0 (7.9–15.4)	0	9.1 (6.1–13.6)	7.8 (4.8–12.3)	10.1 (7.1–14.4)	0.024
TP,g/L	65.6 ± 5.0	64.3 ± 5.3	67.0 ± 4.3	0.016	65.7 ± 5.6	64.6 ± 5.7	66.59 ± 5.34	0.019
ALB,g/L	36.7 ± 3.9	35.33 ± 4.0	38.3 ± 3.0	0	38.0 ± 3.9	36.3 ± 3.8	39.4 ± 3.8)	0
Urea,mmol/L	5.1 (3.8–6.6)	5.1 (3.8–7.1)	4.9 (3.8–6.3)	0.435	5.2 (4.3–6.7)	5.0 (4.1–6.4)	5.3 (4.5–6.8)	0.166
Cr,umol/L	50.0 (43–62.5)	51.0 (43.8–64.2)	49.0 (42–59.5)	0.525	69 (57–81)	60.0 (49.0–76.0)	73.0 (64.3–86.0)	0
UA,umol/L	298.38 ± 106	285.5 (193.8–42)	304.5 (241.3–39)	0.115	355.2 ± 103.6	321.9 ± 91.0	381.6 ± 105.5	0
HGB,g/L	124.5 ± 16.1	120.0 ± 16.2	129.0 ± 14.7	0.007	135.9 ± 19.0	130.5 ± 19.5	140.3 ± 17.4	0
Calf,cm	30.7 ± 3.4	28.2 ± 5.4	33.5 ± 2.3		31.1 ± 3.4	28.6 ± 2.2	34.0 ± 2.1	0
TC,mmol/L	3.9 (3.4–4.7)	3.7 (3.3–4.6)	4.1 (3.6–4.8)	0.106	3.68 ± 0.96	3.7 ± 0.9	3.7 ± 0.9	0.95
TG,mmol/L	1.4 (1.0–1.82)	1.1 (0.99–1.42)	1.6 (1.3–2.3)	0	1.4 (1.1–1.9)	1.3 (1.0–1.68)	1.5 (1.2–2.0)	0.008
HDL,mmol/L	0.99 (0.88–1.3)	0.98 (0.87–1.3)	1.01 (0.88–1.24)	0.977	0.93 ± 0.24	0.9 ± 0.1	0.9 ± 0.2	0.46
LDL,mmol/L	2.4 (1.9–2.9)	2.3 (1.8–2.9)	2.5 (1.98–3.2)	0.144	2.27 ± 0.82	2.2 ± 0.8	2.3 ± 0.8	0.67

*SRS, stroke–related sarcopenia; BMI, Body Mass Index; Duration, time from the stroke onset to hospitalization; TP, total protein; ALB, Albumin; Cr, creatinine; UA, uric acid; HGB, hemoglobin; TC, total cholesterol; TG, triglyceride; HDL, high–density lipoprotein; LDL, low–density lipoprotein. Data presentation: If the continuous data conformed to normal distribution, mean ± SD (standard deviation) was used. If the continuous data did not conform to normal distribution or were in skew distribution, the data were presented as median and interquartile (a 25–75% range)*.

**Table 4:** A categorical variable of patients with and without SRS.

	**Female (*****n*** = **80)**				**male*****(n*** = **179)**			
	**Total**	**SRS**	**Non–SRS**	**P–value**	**Total**	**SRS**	**Non–SRS**	**p**
No.	80	42 (100%)	38 (100%)	0	179	79 (44.1%)	100 (55.9%)	0
Married	61 (76.3)	31 (73.8%)	30 (78.9%)	0.59	18	6 (7.6%)	12 (12%)	0.331
Ischemic	44 (55.0%)	24 (57.1%)	20 (52.6%)	0.685	89	35 (44.3%)	54 (54%)	0.198
Diabetes	19 (23.8%)	10 (23.8%)	9 (23.7%)	0.99	38	18 (22.8%)	20 (20%)	0.651
Hypertension	57 (71.3%)	27 (64.3%)	30 (78.9%)	0.148	136	60 (75.9%)	76 (76%)	0.994
Brainwork	39 (48.8%)	21 (50.0%)	18 (47.3%)	0.814	98	50 (63.3%)	48 (48%)	0.339
Smoking	4 (5%)	2 (4.8%)	2 (5.3%)	0.918	109	65 (82.3%)	44 (44%)	0.068
Alcohol abuse	5 (6.3)	3 (7.1%)	2 (5.3%)	0.729	78	44 (55.7%)	34 (34%)	0.607
Hemiplegia	42 (52.5%)	25 (59.5%)	17 (44.7)	0.186	105	57 (72.2%)	48 (48%)	0.988
Gout	1 (1.3%)	0 (0%)	1 (2.6%)	0.29	28	12 (15.2%)	16 (16%)	0.268
Pneumonia	18 (22.5%)	13 (31.0%)	5 (13.2%)	0.045	55	40 (50.6%)	15 (15%)	0.001
Unable to walk	59 (73.8%)	31 (73.8%)	28 (73.7%)	0.99	107	74 (93.7%)	33 (33%)	0
Cognitive impairment	33 (41.3%)	23 (54.8%)	10 (26.3%)	0.013	83	61 (77.2%)	23 (23%)	0
Aphasia	34 (42.5%)	23 (54.8%)	11 (28.9%)	0.02	94	61 (77.2%)	33 (33%)	0.002
Dysphagia	35 (43.8)	21 (50%)	14 (36.8%)	0.236	85	48 (60.8%)	37 (37%)	0.566
Nasogastric feeding	44 (55%)	29 (69.0%)	15 (39.5%)	0.008	114	75 (94.9%)	39 (39%)	0

*SRS, stroke–related sarcopenia*.

**Figure 1:**
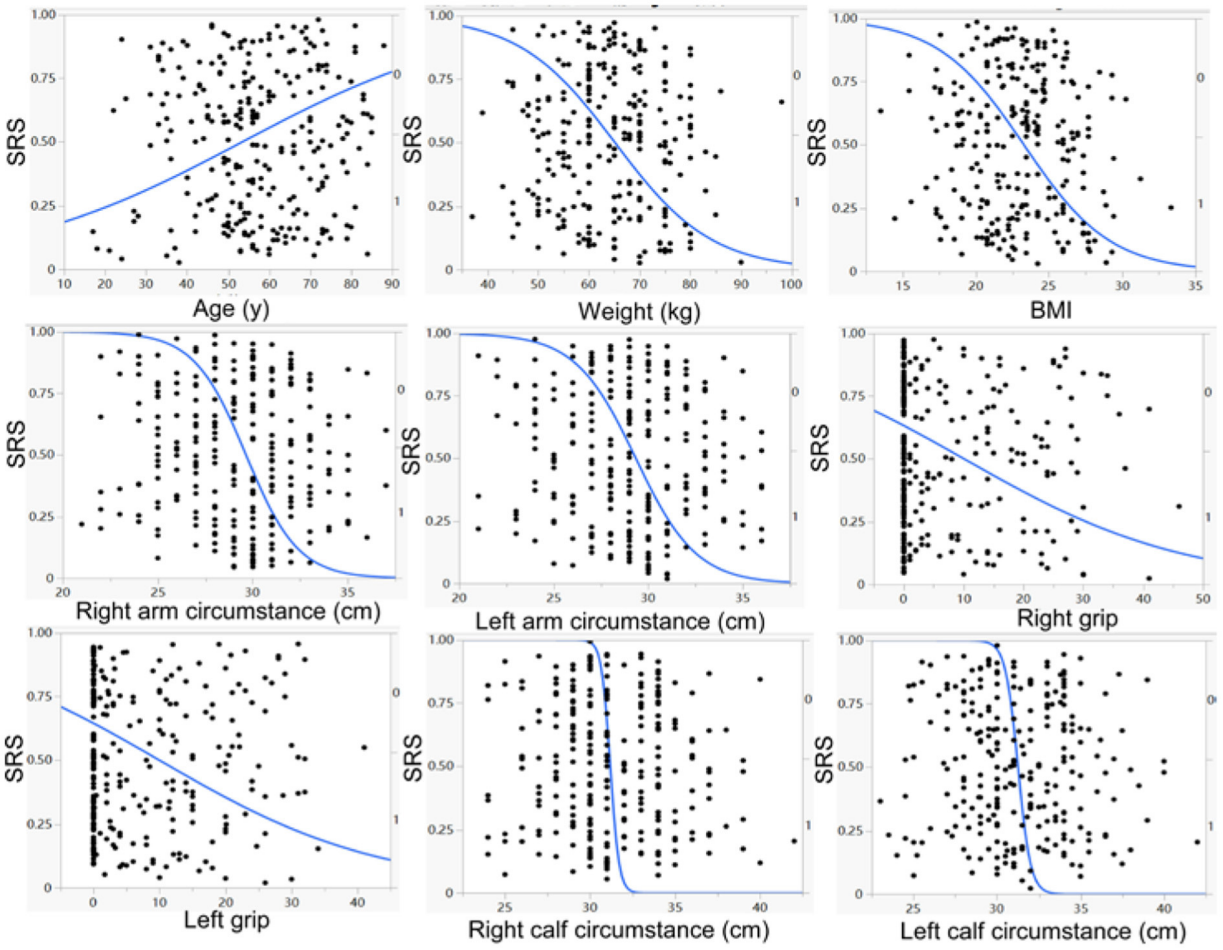
Univariate logistic regression analysis demonstrated a significant (*p* < 0.05) negative correlation of weight, BMI (body mass index) right and left arm circumstances, right and left grips, right and left calf circumstances, but a significant (*p* < 0.05) positive correlation of age, with the prevalence of stroke–related sarcopenia (SRS).

**Figure 2:**
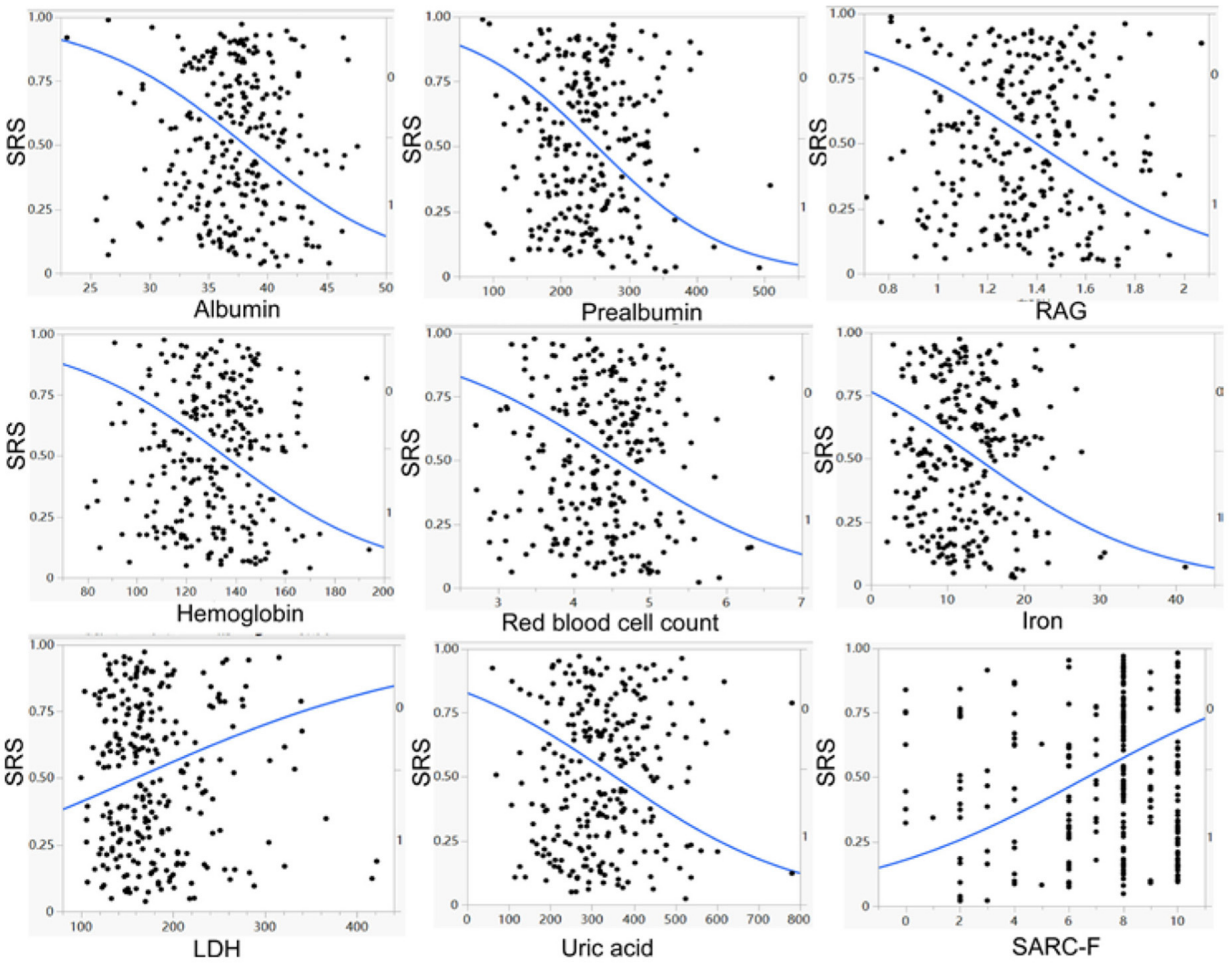
Univariate logistic regression analysis demonstrated a significant (*p* < 0.05) negative correlation of albumin, prealbumin, RAG (ratio of albumin to globulin), hemoglobin, red blood cell count, iron, and uric acid, but a significant (*p* < 0.05) positive correlation of lactate dehydrogenase (LDH) and SARC–F scores with the prevalence of stroke–related sarcopenia (SRS).

No significant (*p* > 0.05) differences were found between patients with and without SRS in sex, height, marriage status, nationality, medical insurance, hemorrhagic stroke, history of coma and tracheotomy, smoking, alcohol abuse, brain surgery, use of thrombolysis, hypertension, diabetes mellitus, gout, bone fracture, depression, anxiety, recovery time, disease course, total serum protein, blood glucose, urea, total cholesterol, triglyceride, HDL, LDL, non-high density lipoprotein cholesterol, and alanine aminotransferase (Tables 1, 2). However, significant (*p* < 0.05) differences were detected by univariate logistic analysis in the NIHSS score, limb muscle strength in the affected side of stroke, the education level of college degree or above, ICU stay, pneumonia, walking ability, cognitive impairment, aphasia, nasogastric feeding, weight, age, left and right upper arm circumferences, left and right grips, left and right calf circumstances, albumin, RAG (the ratio of albumin to globulin), creatinine, uric acid, hemoglobin, prealbumin, iron, LDH, and creatine kinase (Tables 1, 2 and Figures 1, 2). Multivariate logistic regression analysis revealed the following parameters to be significant (*p* < 0.05) risk factors in SRS: college degree or above (OR, 2.1, 95% CI, 1.1–4.1), ICU stay (OR, 1.7, 95% CI, 1.06–2.8), pneumonia (OR, 1.9, 9% CI, 1.1–3.6), walking ability (OR, 2.6, 95% CI, 1.5–4.6), cognitive impairment (OR, 1.8, 95%, 1.1–2.9), aphasia (OR, 2.1, 95% CI, 1.2–3.5), nasogastric feeding (OR, 3.7, 95%, 1.9–7.3), age (OR, 1.04, 95% CI, 1.−1.1), and creatine kinase (OR, 1.1, 95% CI, 0.9–1.2), whereas the following parameters played a protective role in SRS: weight (OR, 0.88, 95% CI, 0.85–0.92), albumin (OR, 0.89, 95% CI, 0.82–0.97), RAG (OR, 0.12; 95% CI, 0.04–0.4), creatinine (OR, 0.994, 95% CI, 0.989–1.), uric acid (OR, 0.996; 95% CI, 0.993–0.998), red blood cells (OR, 0.463, 95% CI, 0.3–0.7), hemoglobin (OR, 0.968; 95% CI, 0.954–0.982), prealbumin (OR, 0.99; 95% CI, 0.986–0.994), and iron (OR, 0.93; 95% CI, 0.89–0.98).

Compared to the patients without SRS, the patients with SRS were significantly (*p* < 0.05) older, less weighed, and had significantly (*p* < 0.05) decreased levels of albumin, RAG, creatinine, uric acid, red blood cell count, hemoglobin, prealbumin, iron, creatine kinase, college education or above, and walking ability. Moreover, more patients with SRS had significantly (*p* < 0.05) longer ICU stay, pneumonia, cognitive impairment, aphasia, and nasogastric feeding than those without SRS.

**Figure 3:**
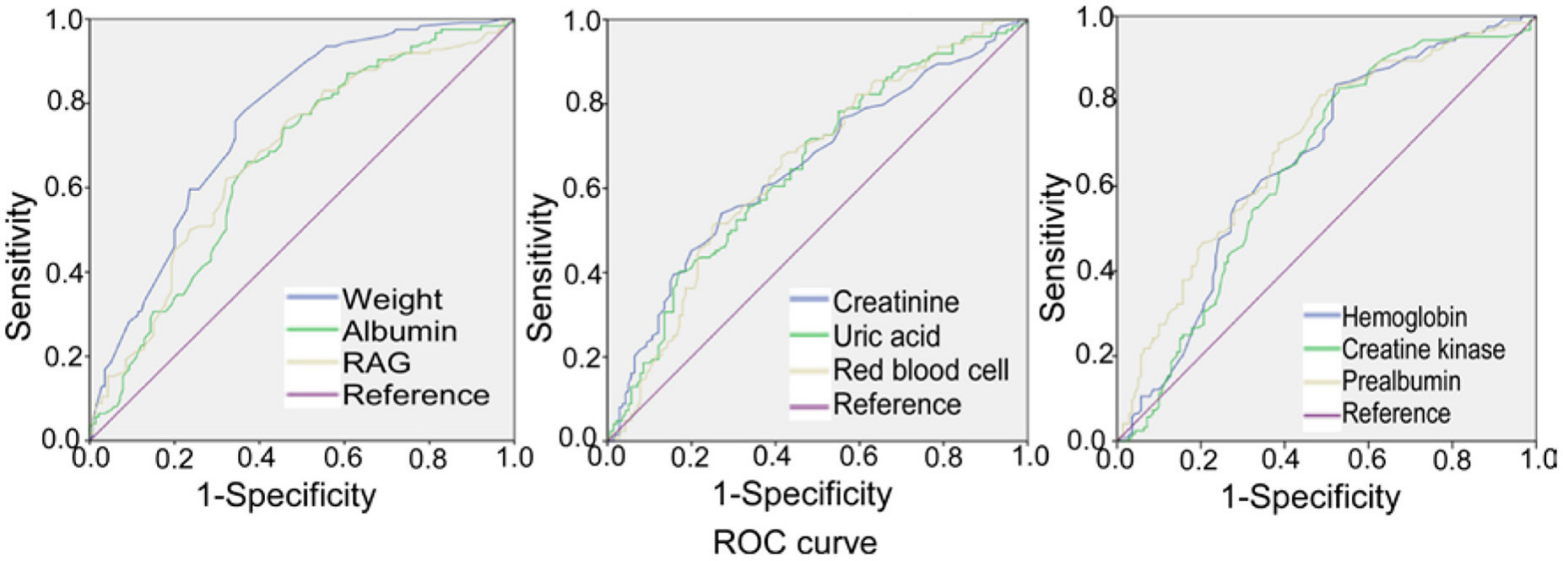
Receiver operating characteristic (ROC) curve analysis of body weight, albumin, RAG (ratio of albumin to globulin), creatinine, uric acid, red blood cell count, hemoglobin, creatine kinase, and prealbumin, with the prevalence of stroke–related sarcopenia.

Receiver operating characteristic (ROC) curve analysis was performed (Table 5 and Figure 3) for the significant measurement parameters in differentiating SRS from non–SRS, and the body weight, albumin, RAG (the ratio of albumin to globulin), creatinine, uric acid, red blood cell count, hemoglobin, creatine kinase, and prealbumin had a sensitivity ranging 0.54–0.863, a specificity ranging 0.386–0.629, and an area under the curve (AUC), ranging 0.649–0.756.

**Table 5:** Receiver operating characteristic (ROC) curve analysis of risk factors.

**Variables**	**Cutoff**	**Sensitivity**	**Specificity**	**Youden index**	**AUC**
RAG	1.29	0.758	−0.536	0.294	0.677
Albumin	37.35	0.661	−0.629	0.290	0.664
Weight	8.95	0.863	−0.386	0.249	0.756
Red blood cell	4.34	0.677	−0.586	0.263	0.651
Uric acid	280.50	0.782	−0.450	0.232	0.649
Creatinine	65.50	0.540	−0.729	0.269	0.649
Prealbumin	234.50	0.702	−0.614	0.316	0.694
Creatine kinase	32.50	0.831	−0.471	0.302	0.646
Hemoglobin	122.50	0.839	−0.479	0.317	0.66

*RAG, Ratio of albumin to globulin*.

The prevalence of SRS was 52.5% (42/80) in women and 44.1% (79/179) in men (Tables 3, 4). Among 179 men, the patients with sarcopenia were significantly older than those without sarcopenia (59.73 ± 12.8 vs. 53.1 ± 13.7 years, *p* < 0.05), but the body weight (61.5 ± 9.7 vs. 70 ± 8.8 kg), serum albumin (36.3 ± 3.8g/L vs. 39.4 ± 3.8g/L), BMI (21.6 ± 3.1 vs. 24.2 ± 2.8 kg/m^2^), TP (64.6 ± 5.7 vs. 66.59 ± 5.34 g/L), TG [1.3 (1.−1.68) mmol/L vs. 1.5 (1.2–2.) mmol/L], and HGB (130.5 ± 19.5 vs. 140.3 ± 17.4 g/L) were significantly lower in patients with than without sarcopenia. Among 80 women, the age of patients with sarcopenia was not significantly different from that of patients without sarcopenia (64.4 ± 16.9 vs. 58.8 ± 16.1 years); however, significant (*p* < 0.05) differences existed in the body weight (52.4 ± 9.1 vs. 63. ± 8.2 kg), serum albumin (35.33 ± 4. vs. 67. ± 4.3g/L), BMI (21. ± 3.4 vs. 25.2 ± 3.3 kg/m), TP (64.3 ± 5.3 vs. 67. ± 4.3 g/L), TG [1.1 (0.99–1.42) vs. 1.6 (1.3–2.3) mmol/L] and HGB (120. ± 16.2 vs. 129. ± 14.7 g/L).

Age (OR, 1.1; 95% CI, 1.−1.2), being unable to walk (OR, 4.9; 95%, 2.6–9.4), coma (OR, 3.1; 95%, 1.7–5.7), pneumonia (OR, 3.1; 95% CI, 1.6–6.3), aphasia (OR, 2.5; 95% CI, 0.4–4.7), and nasogastric feeding (OR, 1.6; 95% CI, 0.9–2.6) were significant (*p* < 0.05) risk factors in sarcopenia in men. BMI (OR, 0.9; 95% CI, 0.8–0.99), body weight (OR, 0.9; 95% CI, 0.8–0.99), TG (OR, 0.5; 95% CI, 0.3–0.8), HGB (OR, 0.9; 95% CI, 0.9–0.99), and serum albumin (OR, 0.8; 95% CI, 0.7–0.9) were significant (*p* < 0.05) protective factors in sarcopenia in men. Nasogastric feeding (OR, 3.4; 95% CI, 1.4–8.6), aphasia (OR, 3.; 95% CI, 1.2–7.5), pneumonia, (OR, 2.9; 95% CI, 1.1–9.3), and cognitive impairment (OR, 2.1; 95% CI, 1.2–4.9) were significant (*p* < 0.05) risk factors, whereas body weight (OR, 0.8; 95% CI, 0.8–0.9), BMI (OR,0.7; 95% CI, 0.6–0.8), HGB (OR, 0.9; 95% CI, 0.9–0.99), and TG (OR, 0.3; 95% CI, 0.1–0.7) were significant (*p* < 0.05) protective factors in sarcopenia in women.

## Discussion

After investigating the prevalence and risk factors of SRS in hospitalized patients receiving rehabilitation treatment, it was found that, compared to the patients without SRS, the patients with SRS were significantly older, less weighed, and had significantly (*p* < 0.05) decreased levels of albumin, RAG, creatinine, uric acid, red blood cell count, hemoglobin, prealbumin, iron, creatine kinase, college education or above, and walking ability besides significantly longer ICU stay, history of pneumonia, cognitive impairment, aphasia, and nasogastric feeding.

To the best of our knowledge, this was the first study to investigate the prevalence, risk factors, and clinical characteristics of SRS in patients with stroke receiving rehabilitation therapy. The prevalence of sarcopenia can be influenced by many factors, including race and age. In China, the prevalence of sarcopenia in individuals aged 60 is 10.6%, with 11.3% in men and 9.8% in women, 9.3% in men and 4.1% in women in Taiwan, and 9.4% among elderly male community dwellers in Hong Kong (22, 23). Older adults with stroke have an accelerated loss in muscle mass and strength compared with those without stroke (24).

The prevalence of SRS was found to be 46.7% in all patients with stroke in the process of rehabilitation in our study, with 44.7% in men and 52.5% in women, which was lower than that of 56% in patients with SRS receiving rehabilitation reported in one study (25). The prevalence of sarcopenia increased with age, and our study confirmed this. The prevalence of SRS was higher in women than in men (52.5 vs. 44.7%), which is in line with the fact that women are less active and carry less outside work (26). The prevalence of SRS of 46.7% in patients receiving rehabilitation in our study was obviously higher than that of 3.1–2.9% in community residents, 14–33% in patients receiving long–term care, and 15–50% in normal old patients of the same age (27).

Our study found that the prevalence of SRS in women was significantly higher than in men, but the age in female patients with sarcopenia was significantly greater than in male patients. This is related to stroke–caused denervation, disuse, remodeling, inflammation, atrophy, and phenotype changes in muscle tissues, subsequently resulting in quick loss of muscle strength and mass. Nerve fiber reinnervation, muscle fiber–type shift, disuse of limb, atrophy, and local inflammatory activation are key features of sarcopenia (28). Sex hormone plays an important role for stroke and sarcopenia. Stroke and sarcopenia mainly occur in 60– to 70–year–old women post menopause and in 50– to 60–year–old men. Estrogen and androgen may improve muscle synthesis (29); however, the secretion of these hormones is significantly decreased in women with menopause and in men of 50–60 years of age, which significantly contributes to the prevalence of SRS in these populations. It has been reported that 20% of men and 5% of women were sarcopenic at the age of 65 years, but up to 50–70% of men and 15% of women were sarcopenic at 85 years (30). However, age and sex were not significantly associated with sarcopenia mainly because stroke is primarily caused by intracerebral hemorrhage or ischemia rather than by age and sex to induce muscle atrophy.

Our findings indicated that weight, BMI, TG, TP, and ALB were significantly lower in patients with than without SRS. Loss of muscle mass and weight but increased fat mass during aging could lead to an increase in the prevalence of sarcopenia (31, 32). The loss of weight mainly results in a decrease in the muscle mass rather than in fatness (33). A cross–sectional study showed that BMI and body weight gradually increased and peaked at 50–59 years in both men and women before decreasing after the age of 60 years (34). However, the obesity prevalence was only decreased a little in adults over the age of 60 years. In a survey (35), the prevalence of obesity was 15.5% in people older than 70 years and 22.9% in people of 60–69 years of age, indicating a little decrease in BMI after the age of 70 years. This means that BMI and body weight decrease while fat increases over 60 years because of muscle atrophy. Hemiplegia results in a long–term disability as the most frequent complication of stroke, and approximately 50% of patients of stroke suffer from hemiparesis, with 30% having difficulty walking (36). Hemiplegia leads to abnormality in muscle metabolism because of denervation, disuse, spasticity, and remodeling, accounting for a complex pattern of skeletal muscle phenotype shift and atrophy. Muscle structural adaptation started as early as 4 h after cerebral infarction, and, a week later, loss of muscles occurred in the limbs. These changes may be caused by disturbed synaptic transmission of muscle–innervating motor neurons, resulting in the reduction of motor unit numbers (12). At the same time, muscle weakness in the limbs develops within 1 week after stroke. These effects are much greater than those caused by age and sex. Weight, BMI, TP, TG, HGB, and serum albumin reflect the body nutrition state, and if patients with stroke had sufficient levels of nutrition state, they would have more energy to resist the enhanced catabolism caused by inactivity and immobilization. In our study, it was found that weight and BMI were negatively associated with SRS, with the prevalence of SRS decreasing as the BMI increased. This suggested that weight and BMI may play a protective role against sarcopenia.

SARC–F scores were risk factors in SRS. SARC–F scores ≥ 4 are liable to have decreased grip strength, slower speed climbing stairs and rising from a chair, and longer walking and falling time (37). Some studies had proved the internal consistence and validity of the SARC–F scores in detecting patients with sarcopenia; however, this scoring system may exaggerate the prevalence of SRS in patients with stroke (38). For patients with hemiplegia, the strength of the paralyzed limb, walking, the rise from a chair, and climbing stairs may all be scored poorly.

The most significant risk factor in sarcopenia was nasogastric feeding as suggested by our study. In fact, some patients with stroke had to use the nasogastric feeding because they had dysphagia problems or were in a coma at the onset of stroke. The size and the location of the stroke lesion are directly associated with dysphagia and may cause swallowing–related muscle atrophy, especially in patients with larger stroke lesions (39). Right hemispheric and brainstem lesions tend to have the pharyngeal dysphagia, but left hemispheric lesions may generate oral dysfunction (21). Moreover, stroke severity is also associated with dysphagia after stroke, with a greater NIHSS score linked to severe dysphagia (39). At the same time, because of slow intestinal peristalsis, dysbiosis, and intolerance to nutrient fluids, patients with stroke are usually accompanied by intestinal malabsorption, leading to malnutrition. Furthermore, dysphagia and placement of a nasogastric tube are significantly associated with aspiration leading to pneumonia, and inflammatory states can also contribute to sarcopenia (21). Thus, protein decomposition is greater than protein anabolism, and a longer time of nasogastric feeding may suggest severe malnutrition to cause reduced muscle mass and strength. Therefore, this vicious circle between dysphagia, malnutrition, and muscle atrophy may result in easy development of sarcopenia. After the stroke onset, malnutrition occurs in 8.2–49.% patients, and dysphagia occurs in 24.3–52.6% (40). Nasogastric feeding was significantly associated with sarcopenia after adjusting for sex, age, NIHSS, the cognitive level, complications, comorbidity, rehabilitation time, and time from the stroke onset to hospitalization (40).

This study had some limitations that should be taken into account when interpreting the consequence. First, this was a case control study, and the prevalence of SRS was only a ratio based on the patients enrolled, which cannot be used as the epidemiological prevalence and incidence in the general population (21). Because of this, the actual prevalence of SRS is not clear in the whole population. Other limitations included a small cohort of patients in an area in China, a one–center study, Chinese patients enrolled only, and non–blindness, which may all affect the generalization of the outcomes. Further prospective studies involving multi–centers and a large area with a larger number of participants over a longer period of time are warranted to confirm the prevalence of SRS.

In conclusion, compared to patients without SRS, patients with SRS are significantly (*p* < 0.05) older, less weighed, and had significantly (*p* < 0.05) decreased levels of albumin, RAG, creatinine, uric acid, red blood cell count, hemoglobin, prealbumin, iron, creatine kinase, college education or above, and walking ability. Moreover, more patients with SRS have significantly (*p* < 0.05) longer ICU stay, pneumonia, cognitive impairment, aphasia, and nasogastric feeding than those without SRS.

## Data availability statement

The original contributions presented in the study are included in the article/supplementary material, further inquiries can be directed to the corresponding author.

## Ethics statement

The studies involving human participants were reviewed and approved by the Ethics Committee of The Second Affiliated Hospital of Kunming Medical University. The patients/participants provided their written informed consent to participate in this study.

## Author contributions

RY was responsible for all the conceptualization, methodology, statistic analysis, the original draft composed, reviewing, and editing. AR, WW, QH, and CX collected and analyzed the data. JO enrolled the participants and collected data. LY was responsible for designing the study and performing data analyses. B–LG revised the original article and reanalyzed the data. All authors contributed to the interpretation of the data and the critical revision and approval of the article. All the authors read and approved the final manuscript.

## Funding

This study was supported by Major Science and Technology Project of Yunnan Province: Medical, Examination, Health and Nursing Service Promotion and Technology Transfer Platform Construction Based on Life and Health (Grant No. 2018ZF016).

## Conflict of interest

The authors declare that the research was conducted in the absence of any commercial or financial relationships that could be construed as a potential conflict of interest.

## Publisher's note

All claims expressed in this article are solely those of the authors and do not necessarily represent those of their affiliated organizations, or those of the publisher, the editors and the reviewers. Any product that may be evaluated in this article, or claim that may be made by its manufacturer, is not guaranteed or endorsed by the publisher.
